# Residential traffic exposure and children's emergency department presentation for asthma: a spatial study

**DOI:** 10.1186/1476-072X-8-63

**Published:** 2009-11-24

**Authors:** Gavin Pereira, AJBM De Vos, Angus Cook

**Affiliations:** 1 School of Population Health M431, The University of Western Australia, 35 Stirling Highway, Crawley WA 6009, Australia; 2 Cooperative Research Centre for Asthma, Sydney, Australia; 3 Telethon Institute for Child Health Research, Centre for Child Health Research, University of Western Australia, 100 Roberts Road, Subiaco WA 6008 Australia

## Abstract

**Background:**

There is increasing evidence that residential proximity to roadways is associated with an elevated risk of asthma exacerbation. However, there is no consensus on the distance at which these health effects diminishes to background levels. Therefore the optimal, clinically relevant measure of exposure remains uncertain. Using four spatially defined exposure metrics, we evaluated the association between residential proximity to roadways and emergency department (ED) presentation for asthma in Perth, Western Australia.

**Method:**

The study population consisted of 1809 children aged between 0 and 19 years who had presented at an ED between 2002 and 2006 and were resident in a south-west metropolitan area of Perth traversed by major motorways. We used a 1:2 matched case-control study with gastroenteritis and upper limb injury as the control conditions. To estimate exposure to traffic emissions, we used 4 contrasting methods and 2 independently derived sources of traffic data (video-monitored traffic counts and those obtained from the state government road authority). The following estimates of traffic exposure were compared: (1) a point pattern method, (2) a distance-weighted traffic exposure method, (3) a simple distance method and (4) a road length method.

**Results:**

Risk estimates were sensitive to socio-economic gradients and the type of exposure method that was applied. Unexpectedly, a range of apparent *protective *effects were observed for some exposure metrics. The kernel density measure demonstrated more than a 2-fold (OR 2.51, 95% CI 2.00 - 3.15) increased risk of asthma ED presentation for the high exposure group compared to the low exposure group.

**Conclusion:**

We assessed exposure using traffic data from 2 independent sources and compared the results of 4 different exposure metric types. The results indicate that traffic congestion may be one of the most important aspects of traffic-related exposures, despite being overlooked in many studies on the exacerbation of asthma.

## Background

There is increasing evidence that residential proximity to major roadways is associated with an elevated risk of asthma exacerbation. A number of epidemiological analyses of the localised impact of traffic emissions have demonstrated associations with airway inflammation and lung function changes, and it has been suggested that these effects may be more detrimental in asthmatics due to their already compromised pulmonary function [1]. Brugge *et al *(2007) reviewed a number of studies conducted in urban areas in the United States and Western Europe, and found a consistent association between asthma and reduced lung function and living near highly trafficked roads [2]. More recently, a consistent association with asthma was found in a review of studies, assessing 'the local impact of traffic' in relation to residential location [3]. In contrast, an earlier review of studies dealing with long-term effects of traffic-related air pollution on the prevalence and incidence of allergic disease and symptoms concluded that the association between traffic-related exposure and asthma and wheezing are consistent in neither adults nor children [4].

The most appropriate method for estimating clinically relevant exposures to traffic emissions has been widely debated. Traffic emissions contain a complex mixture of particulate matter (PM), oxides of nitrogen (NOx), carbon monoxide (CO), oxides of sulphur (SOx), unburned hydrocarbons, and other volatile organic compounds (VOCs)[5]. Because accurate measurement of vehicle pollutants remains problematic, studies have used various measures of exposure to traffic as proxies [6]. Possibly the wide variety of air toxics present in traffic emissions, and their correspondingly variable dispersal with distance, has resulted in lack of consensus on the distance at which their impact diminishes to background 'safe' levels [7]. Interpretation of health impacts is further complicated by the variety of methods that have been developed in an attempt to characterise exposure, ranging from distance to road metrics to the use of distance-weighted traffic counts.

The methods that yield these exposure metrics can be broadly classified into four groups: (1) point pattern methods, (2) traffic counts weighted by distance methods, (3) linear distance methods and (4) methods based on road characteristics. Point pattern methods, such as kernel smoothing, are used to produce continuous estimates of the spatial intensity of the distribution of points across a study area and have been applied to identify clusters of health events [8]. Weighted traffic count methods also produce an interpolated estimate, but these methods make greater use of actual traffic data in the determination of weights. The distance-weighted traffic density (DWTD) approach weights counts using information on how motor vehicle traffic emissions disperse with distance and has been applied in relation to other health events, such as adverse birth outcomes [9]. Linear distance estimates, such as distance to road metrics, are perhaps the most commonly used among spatial studies, and have been applied in the investigation of other asthma outcomes, such as lifetime asthma prevalent asthma and wheeze [10]. Similarly, methods based on road characteristics, such as road length or road length per unit area, have been used as a proxy for residential exposure to traffic [11].

Mixed findings among studies that rely on traffic proximity measures may also be due in part to confounding by socio-economic status (SES). Subjects of low SES may be more likely to live in areas of greater traffic exposure, such as in the United States, but in other settings living closer to the city, and therefore in areas with greater traffic exposure, may be associated with higher SES, such as in many parts of Europe [12].

In this study, we aimed to assess whether exposure to traffic increases the risk of emergency department (ED) presentations in Perth, Western Australia. To provide a comparison in the estimates of effect, we assessed traffic exposure using four different spatial methods (Table 1). The estimates also incorporated two independently derived sources of traffic data: video-monitored traffic counts as well as those obtained from the Main Roads Western Australia (MRWA). The traffic measures applied in this study were selected as they span a range of different spatial characterisations of traffic and therefore result in different interpretations.

**Table 1:** Summary of traffic metrics

	Metric	Type of metric	Data source	Interpretation
1	Kernel density	Point pattern	MRWA	High vs low traffic area based on locations of monitoring sites
2	Distance-weighted traffic density (All traffic, Truck traffic)	Weighted traffic counts	Video monitoring	Vehicle kilometres travelled per peak traffic hour weighted by a pollutant decay function of distance
3	Distance to nearest major road	Linear distance	EDIS MRWA	Distance to nearest road carrying more than 15,000 vehicles per day
4	Road length Road density	Road characteristics		Length of road or density of road within a circular buffer

## Methods

### Study Population and Health Outcomes

#### Study Design

This study is a record-based case-control study using geocoded ED presentation data (2002-2006) for children and young adults, aged 0-19 years, living in the south-western metropolitan area of Perth, Western Australia.

#### Study Area

The study area included 613 census Collection Districts (CDs), encompassing eight Statistical Local Areas, within the south-western region of the Perth metropolitan area. CDs are the smallest available geographical areas for which demographic statistics are disseminated by the Australian Bureau of Statistics, and on average included 225 dwellings. This particular study area was chosen because it is traversed by a combination of both major metropolitan vehicle corridors and lower traffic local roads, and thus provided a reasonable degree of exposure contrast. The total population in the area was 269,734 (2006 Census of Population and Housing). Overall, Perth is a city with low levels of traffic-related air pollution, and a low contribution of industrial air pollution relative to that arising from motor vehicle traffic. The annual mean NO_2 _concentration in 2006 was 0.016 ppm at the Queens Building monitoring station in Perth. The annual mean PM_2.5_concentration in 2006 at Southlake, the closest monitoring station to the study area, was 8.7 μg/m^3 ^respectively [13].

#### Study Population

Cases were individuals aged 0-19 years with residential addresses in the study area, who presented at the ED of any Perth metropolitan hospital between 2002-2006 with a principal diagnosis of asthma (J45) or status asthmaticus (J46). In Australia, access to public hospitals is free, and in metropolitan Perth, these chosen hospitals provide the major avenue for emergency care. For each case, two controls were selected of the same gender and 5-year age category (0-4, 5-9, 10-14, 15-19 years). Controls were similarly defined, except that they attended any of the same group of hospitals with a principal diagnosis of either gastroenteritis (A00-A09) or upper limb injury (S40-S69). Gastroenteritis and upper limb injuries were chosen as control conditions because they had no known or suspected association with proximity to air pollution from motor vehicle traffic. Previous case-control studies have also used gastroenteritis as a control condition in the investigation of air pollution and associated hospitalisation and ED presentation for asthma [14-16].

#### Retrieval of Subject Information

De-identified data for the period 2002-2006 were obtained from the Emergency Department Information System (EDIS). The system draws real time, continuously updated information on ED presentations, including the coded primary diagnosis, from all hospitals across Perth. Only first presentations of cases and controls during the 2002-2006 period were included for the purposes of the analysis.

#### Matching/adjustment variables

Cases and controls were matched by season of presentation at the ED, gender and 5-year age category. We adjusted for ethnicity and SES. Ethnicity was dichotomised between Aboriginal and Torres Strait Islander subjects and others. Due to lack of individual level data, SES was ascertained from the Socio-Economic Index for Areas (SEIFA) Index of Relative Socio-Economic Disadvantage. The SEIFA index is a validated and standardised metric that provides a comparative area-level measure on education, income, occupation, living conditions and access to services [17]. The CD is the smallest aggregate unit for which SEIFA was available. The SEIFA index, corresponding to the census collection district (CD) of the subject's residential address was assigned to the subject record. Next, the SEIFA indices were categorised into quintiles. Two outlier cases and their matched controls were removed from analyses as they exhibited values of SEIFA well below that of the other subjects and were considered to have arisen from a coding error.

### Exposure Metrics

In order to assign an exposure estimate to each case and control, the residential address of each subject was geocoded and subsequently mapped. Next, four different types of metric were used to calculate each subject's exposure at this address using data from two independent sources.

#### Sources of traffic data

**Figure 1:**
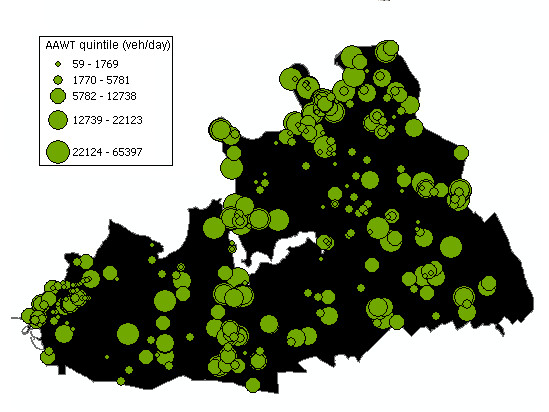
**AAWT quintiles and count site locations for the period 1984-2004**.

Traffic counts and geocoded count site locations were obtained from Main Roads Western Australia (MRWA) for the period 1984 to 2004. To collect these data, automatic traffic recorders were positioned at sites for 1 to 3 days' duration and were supplemented with information from manual count surveys to obtain Annual Average Weekday Traffic (AAWT) counts. The AAWT counts are an estimated average 24-hour traffic volume passing through a site on weekdays, excluding public holidays. The counts were adjusted by season and day of the week. The MRWA site locations were selected because they represent actual or potential locations of traffic congestion, or where major traffic flows occur. Count sites (n = 423) and AAWT quintiles for the period 1984 to 2004 are shown in Figure 1.

As part of the current study, additional information on peak-hour traffic counts were obtained by video monitoring at 102 sites between 8 am and 9 am conducted on weekdays. The monitoring sites encompassed road types that spanned the MRWA Perth Metropolitan Functional Road Hierarchy, defined as Primary Distributor roads (highest traffic), District Distributor A roads, District Distributor B roads, Local Distributors, and Access Roads (lowest traffic). The 102 sites were comprised of sites at 12 Primary Distributors, 30 District Distributors, 25 Local Distributors and 37 Access Roads. The video footage was viewed and motor vehicle movements were counted manually. The average peak-hour traffic (APT) count for each road type was assigned to each road in the study area. A validation study was conducted by filming at 18 MRWA sites, independent of the 102 sites used to obtain the APT values, and showed that the independently collected MRWA traffic counts were strongly correlated with those obtained via video monitoring (Pearson correlation 0.889).

#### Kernel smoothing

Both the AAWT count as well their locations are informative as count sites are more likely to be positioned at highly trafficked locations. Kernel smoothing was applied to these data to calculate two distinct exposure measures as follows. A Bivariate Normal kernel was applied to the site locations, weighted by the site AAWT counts. An empirical estimate of the semivariance range was used to generate the single factor smoothing parameter (1500 m). The kernel scores were dichotomised, creating an exposure variable that reflected high versus low levels of traffic exposure.

#### Distance weighted traffic density

The DWTD measure was selected because it provides a comprehensive measure of local traffic exposure. Furthermore, this measure belongs to a different class of exposure metrics from the others used in this study. Distance weighted traffic density (DWTD) was derived using a method similar to that proposed by Pearson et al [18] and English et al [19] and later applied by Wilhelm et al [9]. DWTD is in fact a distance-weighted traffic volume, rather than a true density (which is defined in terms of traffic per unit area). Pearson's DWTD involves a buffer radius based on a method, which assumes that 96% of the traffic exhaust emissions disperse at 152.4 m (500 ft) from the road according to a Gaussian distribution equation given by

where ***d ***is the shortest distance from the place of residence to the road. Pearson's DWTD was modified to allow traffic along the entire length of road to contribute to a child's exposure estimate, rather than merely the part of the road that passes closest to the place of residence. Each road inside a buffer radius of 228.6 m (700 ft) was divided into 10 m segments. The APT for each segment was weighted by ***w***, multiplied by 10 m and then summed over the buffer to obtain a distance-weighted vehicle-metres travelled per peak hour about the place of residence of that child. These values were later scaled to obtain vehicle-kilometres travelled per peak hour. A separate measure was calculated for truck-only traffic to reflect those exposures more specifically related to diesel emissions, as most light vehicles in Australia do not typically use diesel.

#### Distance to road metrics

The distance to nearest major road was calculated for each subject. Use of this measure assumes that major roads are the greatest contributors to exposure and that the magnitude of exposure is proportional to proximity to this source. A major road was classified as a Primary Distributor according to the MRWA Perth Metropolitan Functional Road Hierarchy [20]. These roads facilitate major regional and inter-regional traffic movement and carry more than 15,000 vehicles per day.

#### Road density metrics

These metrics were used to calculate both total road length and road length per unit area as proxy measures of exposure, and were based on circular "buffers" of radii ranging from 50 m to 400 m in 50 m increments, centred at the subject's residential location. The maximum buffer radius was set at 400 m, more than double the distance at which Pearson assumed majority of emissions dispersed, lest effects extende beyond Pearson's range [18].

### Statistical and computational methodology

Conditional logistic regression was used to calculate risk estimates for presentation with asthma at a hospital ED in relation to each of the traffic exposures. Statistical modelling was implemented using SAS v9.1[21]. Latitude and longitude coordinates of the residential addresses for cases and controls were projected using a transverse Mercator projection into the Geocentric Datum of Australia 1994 Map Grid of Australia Zone 50 using ArcGIS 9.2[22]. All exposure measurements were calculating using ArcGIS 9.2.

### Ethical approval

Ethical approval was obtained from The University of Western Australia Human Research Ethics Committee (RA/4/1/1511) and the Department of Health Western Australia Human Research Ethics Committee (former Confidentiality of Health Information Committee) (#200622).

## Results

### Demographics

A summary of subject characteristics by the matching variables is shown in Table 2 and by other adjustment factors in Table 3. During the study period, 603 subjects (cases) presented at ED with asthma. Therefore, these subjects were matched to 1206 gastroenteritis and upper limb injury controls. The majority of the subjects were male (57%), in the 0-4 age group (37%) and presented at an ED in winter (32%). A small proportion (6%) were Indigenous Australians. The socioeconomic description of the study population is deferred to Section 3.3.

**Table 2:** Summary of matching variables

	Cases		Controls		Total	
	**N**	**%**	**n**	**%**	**n**	**%**

All subjects	603	33	1206	66	1809	100

Gender						
Male	341	57	682	57	1023	57
Female	262	43	524	43	786	43

Age strata						
0-4 years	224	37	448	37	672	37
5-9 years	176	29	352	29	528	29
10-14 years	112	19	224	19	336	19
15-19 years	91	15	182	15	273	15

Season						
Summer	110	18	220	18	330	18
Autumn	172	29	344	29	516	29
Winter	192	32	384	32	576	32
Spring	129	21	258	21	387	21

**Table 3:** Summary of adjustment factors

	Cases		Controls		Total		OR (95% CI)
	**n**	**%**	**N**	**%**	**n**	**%**	

Ethnicity							
Non-indigenous	563 (93)	93	1134 (94)	94	1697 (94)	94	ref
Indigenous	40 (7)	7	72 (6)	6	112 (6)	6	1.12 (0.75, 1.67)
							
SEIFA quintile							
1	74	12	274	23	348	19	ref
2	111	18	266	22	377	21	1.573 (1.12, 2.21)
3	151	25	210	17	361	20	2.979, (2.10, 4.23)
4	142	24	223	18	365	20	2.571 (1.82, 3.63)
5	125	21	233	19	358	20	2.115 (1.50, 2.98)

### Exposure metrics

Means and standard deviations of the four traffic exposure metrics by case and control status are shown in Table 4.

**Table 4:** Means and standard deviations of the four traffic metrics

Road/Traffic Metric	Cases		Controls		All	
	**Mean**	**SD**	**Mean**	**SD**	**Mean**	**SD**

Kernel score indicator for high traffic	NA	NA	NA	NA	NA	NA

Distance-weighted traffic density - All traffic (1000 vehicle km travelled per peak hour of morning traffic)	0.48	0.73	0.64	0.78	0.59	0.77

Distance-weighted traffic density - Truck traffic (1000 truck km travelled per peak hour of morning traffic)	0.02	0.04	0.03	0.04	0.03	0.04

Distance to nearest major road (km)	0.63	0.45	0.59	0.45	0.60	0.45

Length of road within buffer radius (km)						
50 m	0.10	0.04	0.10	0.05	0.10	0.05
100 m	0.44	0.12	0.45	0.13	0.45	0.13
150 m	0.96	0.23	0.98	0.24	0.97	0.23
200 m	1.70	0.36	1.71	0.38	1.70	0.37
250 m	2.63	0.52	2.61	0.55	2.62	0.54
300 m	3.73	0.70	3.70	0.74	3.71	0.73
350 m	5.01	0.89	4.97	0.97	4.98	0.94
400 m	6.45	1.09	6.37	1.21	6.40	1.17

Density of road within buffer radius (km/km^2^)						
50 m	12.90	5.45	13.17	5.59	13.08	5.54
100 m	13.89	3.85	14.37	4.00	14.20	3.96
150 m	13.55	3.21	13.77	3.34	13.70	3.30
200 m	13.48	2.82	13.54	2.99	13.52	2.93
250 m	13.33	2.63	13.26	2.77	13.28	2.72
300 m	13.15	2.46	13.05	2.60	13.09	2.55
350 m	12.97	2.29	12.87	2.51	12.90	2.44
400 m	12.81	2.17	12.64	2.40	12.70	2.33

Results were sensitive to the type of exposure method that was applied (Table 5). The kernel density measure of traffic exposure contrasted high and low traffic exposure based on proximity to a MRWA monitoring site as a proxy for exposure. Traffic exposure, as characterised by this measure, was associated with more than a two-fold increased risk of asthma ED presentation (adjusted OR 2.509, 95% CI 2.001 - 3.146).

**Table 5:** Risk estimates for ED presentation for asthma in children and young adults (0-19 years) in Perth, Western Australia, by using four different Traffic Metrics

Road/Traffic Metric	Unadjusted OR (95% CI)	Adjusted OR* (95% CI)
Kernel score indicator for high traffic	**2.73 (2.19, 3.40)**	**2.51 (2.00, 3.15)**

Distance-weighted traffic density - All traffic (1000 vehicle km travelled per peak hour of morning traffic)	**0.72 (0.62, 0.84)**	**0.73 (0.62, 0.85)**

Distance-weighted traffic density - Truck traffic (1000 truck km travelled per peak hour of morning traffic)	**0.01 (0.00, 0.12)**	**0.01 (0.00, 0.16)**

Distance to nearest major road (km)	**1.24 (1.00, 1.54**)	1.20 (0.96, 1.49)

Length of road within buffer radius (km)		
50 m	0.46 (0.05, 3.90)	0.57 (0.06, 5.03)
100 m	**0.43 (0.19, 0.93)**	0.46 (0.21, 1.01)
150 m	0.76 (0.50, 1.16)	0.74 (0.48, 1.13)
200 m	0.95 (0.73, 1.24)	0.93 (0.71, 1.21)
250 m	1.05 (0.88, 1.26)	1.01 (0.84, 1.22)
300 m	1.06 (0.92, 1.21)	1.03 (0.90, 1.18)
350 m	1.05 (0.94, 1.16)	1.02 (0.92, 1.14)
400 m	1.06 (0.98, 1.16)	1.05 (0.96, 1.14)

Density of road within buffer radius (km/km^2^)		
50 m	0.99 (0.97, 1.01)	0.99 (0.97, 1.01)
100 m	**0.97 (0.94, 0.99)**	**0.97 (0.95, 1.00)**
150 m	0.98 (0.95, 1.01)	0.98 (0.95, 1.01)
200 m	0.99 (0.96, 1.03)	0.99 (0.96, 1.02)
250 m	1.01 (0.97, 1.05)	1.00 (0.97, 1.04)
300 m	1.02 (0.98, 1.06)	1.01 (0.97, 1.05)
350 m	1.02 (0.98, 1.06)	1.01 (0.97, 1.05)
400 m	1.03 (0.99, 1.08)	1.02 (0.98, 1.07)

*Adjusted for ethnicity and SEIFA

The measures of traffic exposure that included a 'length-of-road' component or a 'distance to the nearest major road' component, showed an apparent protective effect on the risk of asthma ED presentation. DWTD_traffic _and DWTD_truck _measures, length of road within 50-400 m buffer measures, and density of road within 50-400 m buffer measures all incorporated a 'length-of-road' component. Each of these exposure types yielded a statistically significant *decrease *in risk of asthma ED presentation.

DWTD_traffic _was associated with a 28% decrease in risk (adjusted OR 0.72, 95% CI 0.62-0.84) per 1000 vehicle kilometres travelled per peak hour of morning traffic. There was also a significant decrease in risk using DWTD_truck _(adjusted OR 0.01, 95% CI 0.00-0.12), but interpretations of this risk estimate is highly problematic as there were very few non-primary roads that were found by video monitoring to have truck traffic. Length of road within 100 m of the residential location was statistically non-significant after adjustment (OR 0.46, 95% CI 0.21-1.01). Density of road within 100 m was associated with a 3.1% decrease in risk (adjusted OR 0.97, 95% CI, 0.95-1.00). In general, there was an apparent increase in risk estimates with increasing buffer size from 50 m to 400 m.

Traffic exposure as characterised by the distance from the place of residence to the nearest main road was also associated with a change in risk of asthma ED presentation. A 1 kilometre increase in the distance to nearest major road was a non-significant increase in adjusted risk (OR 1.20, 95% CI 0.96 - 1.49).

### Socio-economic factors

**Figure 2:**
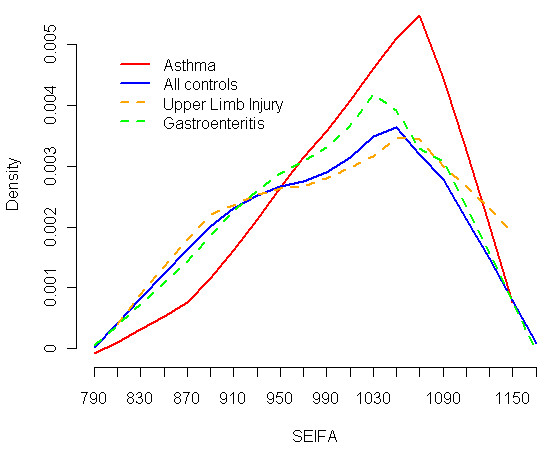
**Density plot of SEIFA index by subject**.

The percentage of cases versus controls in the lowest SEIFA quintile, i.e. the most disadvantaged group, was 12% and 23%, respectively. Of all controls, 23% (n = 274) were in the lowest SEIFA quintile compared to 12% (n = 12) of all cases being in this group (Table 3). A density plot of SEIFA index by subject type is shown in Figure 2. There were proportionally more asthma cases of higher SES than for both gastroenteritis and upper limb injury controls, for whom the SEIFA index values were more evenly distributed. The most disadvantaged group, indicated by the lowest SEIFA quintile, were statistically significantly at less risk of asthma ED presentation than the more advantaged groups. There was a marked 3-fold increase in risk for asthma presentation at an ED (OR 2.98, 95% CI 2.01 - 4.23) for the middle SEIFA quintile compared to the lowest SEIFA quintile group. The change in risk corresponding to an interquartile range increment was also assessed. There was a 75.6% (OR 1.76, 95% CI 1.46 - 2.11) increase in risk per interquartile range increase in the SEIFA index.

### Associations between traffic metrics and socio-economic factors

An increase in the SEIFA index of socio-economic disadvantage (that is, a rise in relative affluence) was associated with a decrease in DWTD for both all traffic and truck measures. An increase in the SEIFA interquartile range was associated with a decrease of 117 (95% CI, -177 - -57) vehicle kilometres per peak hour of morning traffic, and with a decrease of 7 (95% CI, -10 - -3) truck kilometres per peak hour of morning traffic. Both length of road within 100 m and density of road within 100 m also decreased with this interquartile increase in the SEIFA index but were statistically non-significant at this buffer distance (β coefficient for length: -0.003, 95% CI -0.013 - 0.007; for density -0.086, 95% CI -0.396 - 0.225).

Pearson correlations between the length and density buffer metrics with SEIFA index also generally increased with the choice of buffer radius. Correlation between the density buffer metric at 50 m was -0.01285 (p-value = 0.5881), compared with 0.13803 (p-value < 0.0001) at 400 m radius. Additional subanalyses revealed that each increase in the SEIFA interquartile range was associated with a 69 m (69 m, 95% CI 34 - 104) increase in residential distance from the nearest major road.

An interquartile range increase in the SEIFA index was associated with a 66.8% (OR 1.668, 95% CI 1.380 - 2.016) increase in risk of falling within a high traffic area as determined using the dichotomous kernel density measure.

## Discussion

This is the first study to investigate and compare the association between traffic exposure in relation to residential location and the risk of ED presentation for asthma, using a broad range of spatially defined models on the same study population. Although the value of the analysis is the heterogeneity of the exposure measures, a limitation is that the same data was not able to be used within each method. This was not possible due to the inherent differences between the methods: Length and density metrics require the road network as the input. DWTD requires the number vehicles per unit time as an input, which were not available elsewhere, so video-monitoring was conducted. The distance to major road metric requires the Primary Distributor (major road) network as the input. Finally, it is advantageous to have a dense network of sites in the calculation of an exposure metric using a kernel, so we sourced counts from the government traffic authority.

Asthma exacerbation was defined in this study using ED presentation. However, asthma is exacerbated by a variety of means other than vehicle emissions; including lack of asthma management and exposure to allergens. Therefore, asthma ED presentation forms a subgroup of general exacerbation events in the wider population. However, these events are of importance as ED presentation indicates a potentially more severe outcome and are a direct indicator of health service utilization.

Risk estimates varied substantially with the choice of the exposure metric. The main positive result was noted using the kernel density measure of traffic exposure, which contrasted high and low traffic exposure. This metric was associated with more than a two-fold increased risk of asthma ED presentation (adjusted OR 2.51, 95% CI 2.00 - 3.15), although this effect may be partially confounded by socio-economic factors.

The mixed results between exposure metrics may be explained by both the nature of the metric itself as well as the influence of confounding. The 2.5-fold effect corresponding for the kernel score indicator of living in a high traffic area assumes that the locations of the MRWA vehicle count sites serve as reliable proxy indicators for high traffic. However, these sites have been selected, because they represent actual or potential locations of traffic congestion, or locations where major traffic flows occur [23]. Therefore, the kernel effect estimates, which were based on MRWA data, correspond to more extreme contrasts in exposure, and may partially explain the estimated magnitude of the risk. Yet, these effect sizes could also arise if traffic congestion has a greater impact on asthma-related symptoms than traffic flow. This possibility is supported by a 2005 study on infants, which reported an increased prevalence of wheeze (OR 2.5 95% CI 1.15 - 5.42) among those exposed to stop-and-go (bus and truck) traffic compared to unexposed infants [24].

The DWTD, road length and road density measures all incorporated a road length component. The unexpected direction of effect observed using these measures may be partially explained by their statistically significant association with SEIFA, as will be discussed below. The wide 95% CI for DWTD_truck _may be explained by the very small number of trucks being observed during the time windows of video monitoring, particularly in non-primary suburban streets. Indeed, only 40% of subjects had a non-zero value for the DWTD_truck _metric.

Our study indicates that associations between risk of asthma ED presentation and spatially defined traffic exposure proxies may be influenced by SES. Results corresponding to all exposure measures were potentially affected by socio-economic factors. These results also indicate that SES may act to move the risk estimates in either direction to create apparent positive or inverse associations between disease and road/traffic-related exposure variables. An apparently *protective *effect was produced in relation to exposures based on DWTD, road length and density within buffers, and distance to nearest major road. In contrast, SES may have created a spurious *adverse *effect in relation to the dichotomised kernel indicator of residential location near high traffic. Disentangling the directionality of these effects proved a difficult task, particularly for exposure metrics based on length of road and density of road within a particular buffer area. We noted that the odds ratios generally increased with the radius of the buffer (from 50 m to 400 m), and it was important to consider whether the level of socio-economic disadvantage accounted for some of the observed effect. Our supplementary analysis revealed that the SEIFA index increased with the expanding buffer radius, and thus may account for the gradual rise in odds ratios. The inclusion of smaller residential roads in the exposure metrics may have partially contributed to the observed 'protective' effects. We re-analysed the data using a DWTD metric for which only high-traffic roads (Primary and District distributors) were included. However, odds ratios remained below unity, OR 0.988 (0.978, 0.997) per 1000 vehicle kilometer of traffic, per peak hour of morning traffic. The distance to nearest major road metric also produced an effect in this direction which indicates that there are causes of this effect direction other than the inclusion of minor roads.

The distribution of the SEIFA index by health outcome group indicates that ED presentation for asthma is relatively more common for subjects living in the areas of higher SES (Figure 2). In contrast, the gastroenteritis and upper limb injury events are distributed more evenly across the range of SES. Consequently, the matching process may have artificially produced an elevated risk of asthma ED presentation for less disadvantaged subjects beyond that accounted for by adjustment. However, the ratio of controls to cases indicated in Table 3, along with the fact that only 57% of control's had SEIFA values lower than that of their corresponding case, indicates that it is unlikely that the matching process alone could have been responsible for the observed adverse effect of SES. The use of area-level estimates for SES may incompletely characterise this effect, thus highlighting the potential of an ecological fallacy. We expected the opposite relationship given that asthma is potentially more likely to be clinically unstable among children from lower socio-economic backgrounds. In other studies, the financial cost of asthma treatment can induce difference in access across socio-economic groups. However this factor is of relatively low influence in Australia where the federal government's Pharmaceutical Benefits Scheme heavily subsidises medication and Medicare subsidises consultation to the extent that the very low SES population's GP consultations are fully bulk-billed. Repeating the full analysis by matching on SEIFA quintile revealed that risk estimates only changed slightly.

Further investigation is required into the association between an increase in SEIFA and emissions in the local context, including the role of industrial pollutants. Despite these potential hazards, most of the criteria air pollutants in these suburbs are derived from traffic sources. The study area has limited industrial activity and is primarily residential. Nonetheless, there remains a potential small effect related to differential residential exposure to additional hazards across socioeconomic groups.

Finally, we note that statutory air monitoring in Perth is conducted at very few locations, none of which were located in the study area over the study period. Consequently, like many other studies that have assessed the effect of traffic exposure on asthma exacerbation, we modelled exposure in a spatial context, and our exposure measures can therefore be considered 'cross-sectional'. We note that only wide-area pollutant data was available for our project, and if used would raise the likelihood of significant exposure misclassification. By using purely spatially defined proxies, our exposure metrics also lack direct dose and temporal information. Nonetheless, the exposure measures used in this study have some distinct advantages. Firstly, the exposure is directly related to the inherent focus of the research question, namely 'residential exposure to traffic', which requires a proximity component as well as a traffic exposure component. Secondly, our exposure metrics focus on motor vehicles themselves and incorporate the entire mixture of associated putative hazards (such as tyre and road particulate matter, non-combusted fuel vapours and the inherent mixture of emission toxicants). Finally, exposures defined in this way may be more relevant for the development of suitable community health interventions, such traffic control measures being incorporated into urban planning of residential areas.

## Conclusion

We assessed the risk of asthma exacerbations, as defined by hospital ED presentation for asthma, in relation to residential exposure to motor vehicle traffic. The strength of our study was that we were able to assess and compare exposure using four different metrics, and traffic data from two independent sources. Our analyses highlights the potential confounding of spatially designed traffic exposure metrics by geographical gradients in SES. Our results also indicate that traffic congestion may be one of the most important aspects of traffic-related exposures, despite being overlooked in many studies on the exacerbation of asthma. The identification and quantification of air toxics, that may cause the exacerbation of asthma, is beyond the scope of this record-based case-control study. Yet, the results of this study serve to increase our understanding of the association between residential proximity to traffic and the risk of ED presentation for asthma in children and young adults.

## Competing interests

The authors declare that they have no competing interests.

## Authors' contributions

GP carried out the spatial and statistical analyses, including exposure calculation, interpreted the results, and wrote the manuscript. AJBMDV conducted the sample selection and helped revise the manuscript. AC provided input into the interpretation of the results and also helped revise the manuscript. All authors declare that they have read and approve the final manuscript.
